# Diabetic Ketoacidosis Complicated by Cerebral and Pulmonary Oedema in an Adult

**DOI:** 10.7759/cureus.104796

**Published:** 2026-03-06

**Authors:** Navitha Singh, Zeyn Mahomed, Kgalalelo Molefe, Nabeelah Nalla, Peter Beskyd, Deidre Hoffman, Anzanne Du Preez, Faatimah Dollie, Mohammad H Khan, Juwairiah Jassat

**Affiliations:** 1 Emergency Medicine, Chris Hani Baragwanath Academic Hospital, Johannesburg, ZAF; 2 Emergency Medicine, University of the Witwatersrand, Johannesburg, Johannesburg, ZAF

**Keywords:** diabetic keto acidosis, diffuse cerebral edema, fluid resuscitation, medical intensive care unit (micu), non-cardiogenic pulmonary edema

## Abstract

Diabetic ketoacidosis (DKA) is a life-threatening metabolic emergency. While cerebral and pulmonary oedema are recognised complications in paediatric populations, they are exceptionally rare and poorly characterised in adults. This case report describes an adult patient with severe DKA who developed both cerebral and pulmonary oedema during treatment.

A previously healthy 36-year-old man presented with a two-week history of polyuria and polydipsia and progressive dyspnoea. He was alert and tachycardic and exhibited Kussmaul respirations. Initial investigations confirmed severe DKA: serum glucose 22.1 mmol/L, bicarbonate <4 mmol/L, and an elevated anion gap. Point-of-care ultrasound (POCUS) confirmed volume depletion. Management followed standard DKA protocol with cautious fluid resuscitation and intravenous insulin.

At 11 hours post-presentation, after receiving 4 litres of fluid, the patient developed acute respiratory distress and a decline in Glasgow Coma Scale (GCS) to 12. Imaging confirmed non-cardiogenic pulmonary oedema and cerebral oedema with leptomeningeal enhancement. He was intubated, required inotropic support, and was transferred to the intensive care unit (ICU). Management included continued DKA treatment, dexamethasone for cerebral oedema, empirical antibiotics, and strict fluid regulation. The pulmonary oedema resolved, and a repeat computed tomography (CT) scan of the brain showed resolution of cerebral oedema. The patient was successfully extubated on day 6 and made a complete neurological recovery.

This case highlights that cerebral and pulmonary oedema, though rare, can occur in adults with DKA. It underscores the need for vigilant monitoring for these complications even with standard, cautious management. A multifactorial pathophysiology is likely, requiring an integrated critical care approach for successful outcomes. Emergency and critical care practitioners should be aware of these potential life-threatening complications in adult DKA.

## Introduction

Diabetic ketoacidosis (DKA) is a life-threatening hyperglycaemic emergency caused by absolute or relative insulin deficiency. This deficiency impairs cellular glucose uptake and prevents the inhibition of hepatic gluconeogenesis and glycogenolysis, leading to profound hyperglycaemia. Elevated intravascular glucose concentrations trigger osmotic diuresis, leading to significant fluid and electrolyte losses and ultimately dehydration [1].

Concomitantly, insulin deficiency, in association with elevated levels of counterregulatory hormones (glucagon, cortisol, catecholamines, and growth hormone), stimulates lipolysis and hepatic ketone body production, leading to a high-anion-gap metabolic acidosis (HAGMA). This acidotic state promotes a transcellular shift of hydrogen ions into cells, in exchange for potassium, often resulting in pseudo-hyperkalaemia despite total body potassium depletion [2].

The cornerstone of DKA management includes prompt and careful fluid resuscitation, cautious correction of potassium abnormalities, intravenous insulin therapy, and identification and treatment of the underlying precipitant [3]. Although most cases resolve with appropriate therapy, a subset of patients may develop serious complications during treatment. These include hypoglycaemia, hypokalaemia, cerebral oedema, and non-cardiogenic pulmonary oedema.

Although cerebral and pulmonary oedema are well documented in paediatric patients with DKA, they remain rare but potentially fatal complications in adults [4,5]. A literature search revealed isolated and infrequent case reports [6,7]. Their pathophysiology, optimal management, and outcomes in the emergency and critical care settings are not well described. This case report highlights an adult patient presenting with severe DKA whose clinical course was complicated by both cerebral and pulmonary oedema. The case aims to contribute to the limited body of literature on these complications and to inform emergency medicine and critical care practice.

## Case presentation

A 36-year-old African man with no known comorbidities or prior medical history presented to the emergency department (ED) with a two-week history of polyuria, polydipsia, and progressive dyspnoea. He denied symptoms of fever, cough, vomiting, diarrhoea, chest pain, or headache. There was no history suggestive of toxin ingestion or use of traditional medicines. His social history was notable for recreational alcohol use, though he reported no recent consumption.

**Figure 1:**
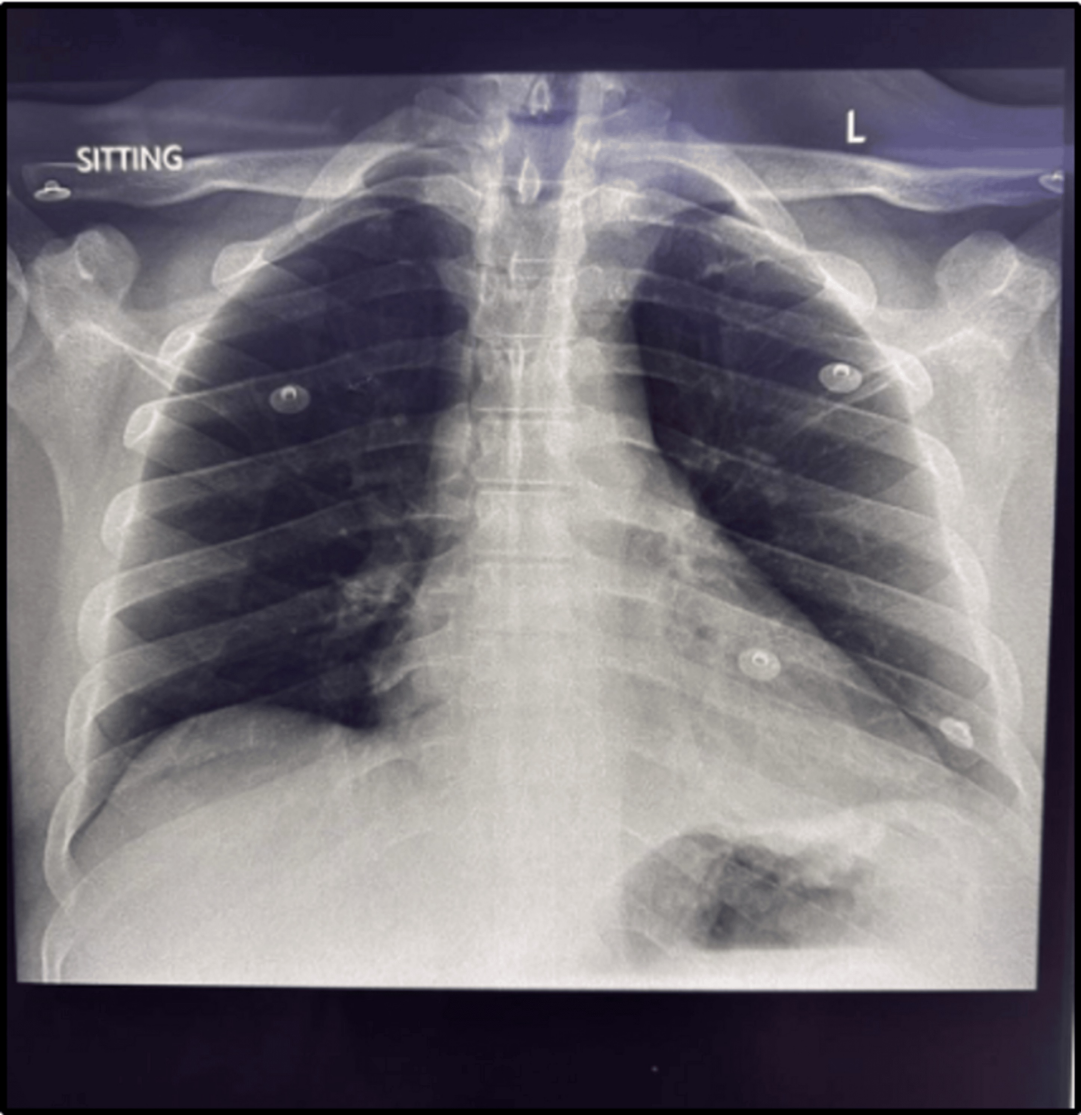
First chest radiograph

On examination, he was awake and alert, maintaining his airway independently. He exhibited increased respiratory effort with Kussmaul breathing, but oxygen saturation remained within normal limits on room air. He was haemodynamically stable, with a sinus tachycardia of 112 beats per minute and a blood pressure of 140/78 mmHg. Perfusion appeared adequate, and there were no signs of clinical shock. Neurologically, the patient was fully oriented with equal, reactive pupils and no focal deficits. There were no signs of meningism. Ear, nose, and throat as well as dermatological examinations were unremarkable. An initial chest X-ray (Figure 1) was normal. The first venous blood gas (Table 1) revealed severe HAGMA (anion gap=29), with a partially compensated respiratory alkalosis.

**Table 1:** Initial venous blood gas with accompanying interpretation pCO₂: partial pressure of carbon dioxide; pO₂: partial pressure of oxygen; HCO₃⁻: bicarbonate; BE: base excess; Na⁺: sodium; K⁺: potassium; Cl⁻: chloride; AG: anion gap; HAGMA: high-anion-gap metabolic acidosis

Parameter	Patient result	Reference range	Unit	Interpretation
pH	6.95	7.31-7.41	-	Severe acidosis
pCO₂	18	38-50	mmHg	Respiratory compensation (Kussmaul breathing)
pO₂	35	30-50	mmHg	Venous blood gas
HCO₃⁻	3.5	22-28	mmol/L	Profoundly low (severe metabolic acidosis)
BE	-25	-2 to +2	mmol/L	Severe base deficit
Lactate	3.2	<2.0	mmol/L	Mildly elevated (due to hypoperfusion/hypovolaemia)
Na⁺	132	135-145	mmol/L	Hyponatraemia (often dilutional/pseudohyponatraemia due to hyperglycaemia)
K⁺	3.2	3.5-5.0	mmol/L	Mild hypokalaemia
Cl⁻	95	98-110	mmol/L	Relatively low
Glucose	22.1	4.0-6.0	mmol/L	Severe hyperglycaemia
AG	29	8-12	mmol/L	HAGMA (calculated: Na-(Cl+HCO₃))

Using point-of-care ultrasound (POCUS), the authors examined the heart and found normal chamber size, an underfilled left ventricle, and hyperdynamic function, with a grossly preserved ejection fraction. There was no valvular pathology or signs of right heart strain. The inferior vena cava exhibited more than 50% collapsibility, which is consistent with intravascular volume depletion. There was bilateral apical lung sliding with an A-line profile and no B-lines or effusions visualised. Bilaterally normal renal morphology was evident. The femoral and popliteal veins were compressible bilaterally with no sonographic evidence of deep vein thrombosis. A computed tomography (CT) scan of the brain was not clinically indicated at the time of presentation and therefore was not performed. The results of the initial investigations are shown in Tables 2-4.

**Table 2:** Biochemistry and electrolytes eGFR: estimated glomerular filtration rate

Test	Result	Reference range
Sodium	131 mmol/L	136-145 mmol/L
Potassium	3.4 mmol/L	3.5-5.1 mmol/L
Chloride	92 mmol/L	98-107 mmol/L
Bicarbonate	<4 mmol/L	22.1-28.3 mmol/L
Urea	4.4 mmol/L	2.1-7.1 mmol/L
Creatinine	93 µmol/L	64-104 µmol/L
eGFR	92 mL/min/1.73 m²	-
Calcium	2.38 mmol/L	2.15-2.50 mmol/L
Magnesium	0.92 mmol/L	0.63-1.05 mmol/L
Phosphate	1.44 mmol/L	0.78-1.42 mmol/L

**Table 3:** Full blood count WCC: white blood cell count; RBC: red blood cell count; MCV: mean corpuscular volume; MCH: mean corpuscular haemoglobin; MCHC: mean corpuscular haemoglobin concentration; RDW: red cell distribution width

Test	Result	Reference range
WCC	13.24×10⁹/L	3.92-10.40×10⁹/L
RBC	4.95×10¹²/L	4.19-5.85×10¹²/L
Haemoglobin	14.9 g/dL	13.4-17.5 g/dL
Haematocrit	0.443 L/L	0.390-0.510 L/L
MCV	89.5 fL	83.1-101.6 fL
MCH	30.1 pg	27.8-34.8 pg
MCHC	33.6 g/dL	33.0-35.0 g/dL
RDW	13.6%	12.1-16.3%
Platelets	426×10⁹/L	171-388×10⁹/L

**Table 4:** Liver function, pancreatic enzymes, and other investigations ALP: alkaline phosphatase; GGT: gamma-glutamyl transferase; ALT: alanine aminotransferase; AST: aspartate aminotransferase

Test	Result	Reference range
Total bilirubin	4 µmol/L	5-21 µmol/L
Conjugated bilirubin	<3 µmol/L	0-3 µmol/L
Total protein	80 g/L	60-78 g/L
Albumin	37 g/L	35-52 g/L
ALP	153 U/L	53-128 U/L
GGT	61 U/L	<68 U/L
ALT	32 U/L	10-40 U/L
AST	24 U/L	15-40 U/L
Lipase	31 U/L	0-160 U/L
Amylase	51 U/L	23-85 U/L
C-reactive protein	58 mg/L	<10 mg/L
Thyroid-stimulating hormone	1.34 mIU/L	0.35-5.50 mIU/L
Blood cultures	No growth of aerobic or anaerobic organisms after 5 days	-
Urine dipstick	Positive for glycosuria and ketonuria	-

The patient was diagnosed with severe DKA, and resuscitative measures were promptly initiated. Initial management involved correction of hypokalaemia with intravenous potassium replacement to achieve a serum potassium level >3.5 mmol/L, followed by initiation of a continuous intravenous infusion of short-acting insulin at 0.1 IU/kg/hour. Isotonic crystalloid resuscitation with Plasma-Lyte was administered under ultrasound guidance. A maintenance infusion containing potassium chloride and magnesium sulphate was subsequently commenced to maintain serum potassium between 3.5 and 5.5 mmol/L. Additional interventions included high-dose intravenous thiamine, subcutaneous long-acting insulin, and enoxaparin for venous thromboembolism prophylaxis. Broad-spectrum antibiotics (1.2 g amoxicillin-clavulanic acid) were initiated empirically.

The patient was monitored in a high-care area, with blood gas analysis performed every 2-4 hours. Judicious fluid administration continued throughout his ED stay, with POCUS used to guide volume resuscitation. At 11 hours post-presentation, the patient had received a cumulative total of 4 litres of intravenous fluid, comprising an initial bolus of 20 mL/kg in the first hour, followed by a maintenance infusion at 1.5 mL/kg/hour, with 250 mL aliquots of fluid guided by serial POCUS assessments. Despite the administered volume, POCUS findings continued to suggest intravascular depletion.

**Figure 2:**
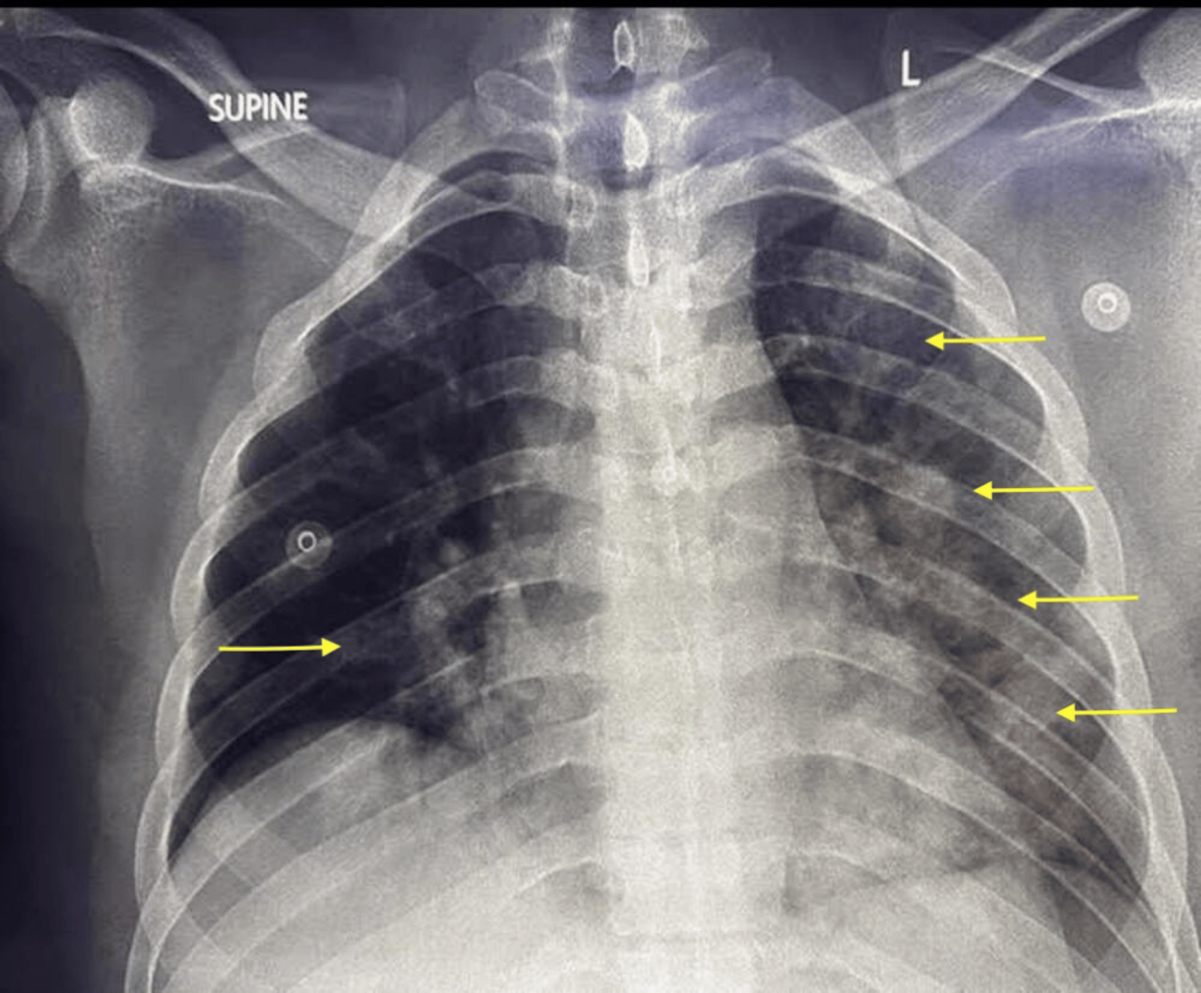
Follow-up chest radiograph indicating pulmonary oedema. The yellow areas indicate new hazy opacificities

The patient then developed increased work of breathing. On auscultation, bilateral coarse crepitations were noted, and POCUS demonstrated new bilateral confluent B-lines with preserved lung sliding, suggestive of pulmonary oedema. A focused echocardiogram revealed a hyperdynamic left ventricle with preserved chamber sizes and valvular function. A repeat chest X-ray demonstrated bilateral infiltrates, more prominent on the left (Figure 2). Despite these radiological and sonographic changes, the patient remained normoxic (SpO₂ 96% on room air) and normocapnic on arterial blood gas analysis. In response, the maintenance fluid rate was reduced from 1.5 to 1 mL/kg/hour.

Concurrently, neurological status declined, with the patient's Glasgow Coma Scale (GCS) dropping to 12/15 (E4V3M5). A CT scan of the brain revealed evidence of cerebral oedema, without basal cistern effacement or herniation. Osmotherapy with mannitol or hypertonic saline was deferred, and diuretics such as furosemide were withheld due to ongoing concern for intravascular volume depletion.

DKA management continued without interruption, with an improving acid-base status noted on serial blood gas analyses. However, in response to deteriorating neurological function and progressive acidosis, the patient was intubated following consultation with the critical care team. Ketamine was used as the induction agent, with suxamethonium for neuromuscular blockade. Fentanyl was administered adjunctively to blunt sympathoadrenal responses during laryngoscopy. Post-intubation, the patient developed hypotension and worsening acidosis, necessitating the initiation of inotropic support and the administration of 50 mL of 8.5% sodium bicarbonate.

Following initial stabilisation, the patient was transferred to the intensive care unit (ICU). In the ICU, the patient remained sedated and mechanically ventilated. Antibiotic therapy was continued, and enteral nutrition was initiated early via a nasogastric tube. Dexamethasone was administered to address the cerebral oedema. Total fluid intake was tightly regulated at 110 mL/hour, while intravenous maintenance fluids were gradually tapered as enteral feeds were increased. The Insulin infusion was progressively down-titrated as glycaemic control improved.

During the ICU stay, the patient developed hypernatraemia (serum sodium 161 mmol/L). This was corrected to 145 mmol/L over 72 hours via careful enteral free-water administration via the nasogastric tube, while total fluid administration remained restricted. Pulmonary oedema resolved, and the patient was successfully extubated on ICU day 6. Glycaemic control was transitioned to a subcutaneous sliding-scale insulin regimen. A repeat CT scan of the brain demonstrated resolution of cerebral oedema and leptomeningeal enhancement. Neurologically, the patient returned to a GCS of 15/15 and was subsequently stepped down to a general medical ward in stable condition.

## Discussion

This case describes an adult patient with DKA whose clinical course was complicated by the sequential development of two rare but serious conditions: cerebral oedema and non-cardiogenic pulmonary oedema. While DKA is a common endocrine emergency, the concurrent manifestation of these complications in an adult is exceptionally infrequent and carries a high risk of morbidity and mortality. This discussion will explore the diagnosis, pathophysiology, and management challenges posed by each, highlighting the critical intersection of their treatment strategies.

DKA is diagnosed by the characteristic triad of hyperglycaemia, ketonaemia, and HAGMA. In this case, early recognition and protocol-driven management were promptly initiated. However, despite appropriate therapy, the patient's course deviated significantly, underscoring that life-threatening complications can arise even with standard care.

Cerebral oedema is a well-recognised and devastating complication of DKA, most frequently described in the paediatric population, where it remains a leading cause of DKA-related mortality. Its occurrence in adults, while less common, is increasingly acknowledged and carries a similarly grave prognosis. Clinically, it should be suspected in any patient with a deteriorating level of consciousness, new-onset headache, persistent vomiting, or focal neurological signs. Recognised risk factors, largely extrapolated from paediatric studies but applicable to young adults, include the following: age <25 years, new-onset diabetes, prolonged duration of symptoms, severe acidosis (pH <7.0 or bicarbonate <5 mmol/L), hypocapnia (a low pCO₂ as a compensatory response), high presenting serum osmolality (>330 mOsm/kg), and a failure of the serum sodium to rise appropriately as glucose falls during treatment [1].

In our patient, a drop in GCS from 15 to 12 was the critical clinical trigger that prompted urgent action. Neuroimaging is paramount in this context, not only to confirm cerebral oedema but also to exclude alternative or contributing intracranial pathologies, such as thrombosis, infection, or haemorrhage. The CT findings of cerebral oedema, accompanied by leptomeningeal enhancement, raised the dual possibility of neurosepsis. Consequently, empirical antibiotics were initiated while the culture results were pending. A lumbar puncture was appropriately deferred due to the significant risk of cerebral herniation in the setting of established oedema and mass effect.

The precise pathophysiology of cerebral oedema in DKA, particularly in adults, remains an area of active investigation. The traditional, and once widely accepted, osmotic theory proposed that rapid intravenous fluid administration caused a swift decline in serum glucose. If this decline outpaced the brain's ability to clear its own accumulated intracellular osmolytes (such as sorbitol and myo-inositol), a dangerous osmotic gradient would be created, driving water into brain cells [2,3]. While conceptually elegant, this theory has been challenged, notably by the landmark Pediatric Emergency Care Applied Research Network (PECARN) FLUID trial. This large, multicentre trial found no association between the rate of fluid administration and the risk of cerebral oedema or neurological decline in children with DKA [4].

The theory has now shifted the focus toward a more complex, multifactorial model. Current understanding suggests an initial insult from cerebral hypoperfusion, leading to cytotoxic oedema and the generation of inflammatory mediators. Subsequent fluid resuscitation and reperfusion may then trigger a second wave of injury, characterised by increased cerebral blood flow and disruption of the blood-brain barrier, resulting in vasogenic oedema [5,8]. This "two-hit" hypothesis better accounts for the timing of neurological deterioration, which often occurs several hours into treatment, as seen in this patient.

This evolving pathophysiological understanding creates a therapeutic dilemma. While the PECARN trial suggests that overly cautious fluid resuscitation may not prevent cerebral oedema, the risk of exacerbating cerebral oedema with aggressive fluids remains a theoretical concern. Therefore, a balanced, cautious approach to fluid resuscitation is still widely advocated, particularly in patients with multiple risk factors, and this was the strategy adopted in this case.

The use of dexamethasone in this context was a novel, pathophysiology-driven intervention. Its well-established role in reducing vasogenic oedema associated with brain tumours and traumatic brain injury, by stabilising the blood-brain barrier, provided the rationale for its use here. While its efficacy in DKA-associated cerebral oedema is unproven, it may offer a targeted approach to the proposed vasogenic component of the oedema.

The decision to withhold hyperosmolar agents like mannitol or hypertonic saline was a considered one. These agents are typically reserved for patients with signs of impending or active cerebral herniation, where their rapid onset of action can be lifesaving. In this case, with a stable, though reduced, GCS and no herniation syndromes, the potential risks were deemed to outweigh the potential benefits. The cornerstone of management, therefore, became the rigorous application of general neuroprotective principles: head-of-bed elevation to 30-45°, strict maintenance of normoxia, normocarbia (avoiding both hypocapnia and hypercapnia, which can affect cerebral blood flow), normotension, and normoglycaemia.

The development of acute respiratory failure in DKA is an uncommon but ominous sign, associated with significantly increased morbidity and mortality [9,10]. The differential diagnosis is broad, including community-acquired pneumonia, acute respiratory distress syndrome (ARDS) secondary to pancreatitis or sepsis, and pulmonary oedema. In this patient, the acute onset of respiratory distress, the absence of clinical or radiographic signs of infection, and normal pancreatic enzymes made pneumonia and pancreatitis-associated ARDS less likely. The clinical picture, supported by POCUS findings of bilateral confluent B-lines and a chest radiograph showing pulmonary oedema, pointed towards a non-cardiogenic cause. This was confirmed by a formal echocardiogram, which excluded left ventricular systolic or diastolic dysfunction.

The pathogenesis of non-cardiogenic pulmonary oedema in DKA is thought to arise from two primary, potentially overlapping mechanisms [11]. The first is hydrostatic or osmotic oedema. Profound hyperglycaemia creates a powerful osmotic gradient that draws fluid from the intracellular space into the extracellular compartment. This can transiently increase central blood volume and pulmonary venous pressure, leading to hydrostatic pulmonary oedema. This mechanism typically presents early, often before treatment is initiated, and tends to resolve with gradual glucose correction and the subsequent fall in plasma osmolality. Concomitant renal impairment, which hampers the excretion of this fluid load, is a common contributing factor. The patient in this case had normal renal function, making this mechanism less likely.

The second mechanism is increased permeability or non-hydrostatic oedema: This represents a true "capillary leak" syndrome. Chronic hyperglycaemia is known to cause microangiopathic changes, including thickening of the alveolar epithelial and pulmonary capillary basement membranes. This may predispose patients to increased endothelial permeability. The inflammatory milieu of DKA, characterised by elevated cytokines and oxidative stress, can then act as a trigger, increasing pulmonary vascular permeability and allowing protein-rich fluid to leak into the interstitial and alveolar spaces [11]. This type of oedema typically presents later in the treatment course, as in our case, and is less responsive to simple glucose correction.

The timing of our patient's deterioration (occurring several hours into treatment, with preserved renal function and initially clear lung fields) strongly favours a permeability-mediated mechanism. The concurrent presence of cerebral oedema also raises the possibility of neurogenic pulmonary oedema (NPE). NPE is a well-described phenomenon following a profound neurological insult (e.g., seizure, traumatic brain injury, subarachnoid haemorrhage) that triggers a massive catecholamine surge. This surge causes acute pulmonary venoconstriction and increased capillary permeability. While an attractive hypothesis, the absence of a clear, sudden neurological trigger in our patient made NPE a less probable explanation.

The management of DKA-associated pulmonary oedema is largely supportive. The fundamental principle is to treat the underlying DKA while providing respiratory support. Invasive mechanical ventilation is generally avoided if possible, due to the risks of hypotension from induction agents and the potential for ventilator-associated lung injury. However, indications for intubation include impending respiratory failure, severe hypoxaemia, or, as in this case, the inability to protect the airway or maintain adequate ventilation in a patient with concurrent cerebral oedema. In similar case reports, management has remained largely supportive [6,7].

The intubation itself was a high-risk procedure, managed with a haemodynamically favourable rapid sequence induction (ketamine for its sympathomimetic properties and suxamethonium for its rapid offset) and fentanyl to blunt the sympathetic response to laryngoscopy. Post-intubation hypotension was anticipated and managed with inotropic support. Mechanical ventilation was strategically targeted to preserve some spontaneous respiratory effort, which can improve venous return and ventilation-perfusion matching, while ensuring that the patient's compensatory respiratory drive for the metabolic acidosis was not completely suppressed.

The decision-making around fluid management was complex. Diuretics were appropriately withheld, as there was no clinical or sonographic evidence of frank volume overload. This patient was not fluid-overloaded systemically, but rather had a pathological leak in the pulmonary circulation. Conversely, fluid resuscitation was carefully continued but at a cautiously controlled rate, mindful of the dual risks. These dual risks are under-resuscitation, which prolongs DKA, and over-resuscitation, which can worsen both cerebral and pulmonary oedema. The rate was kept within the cautious parameters (<50 mL/kg in the first hour), often recommended in paediatric guidelines to mitigate the risk of cerebral oedema [12]. This demonstrates how the management of one complication directly informed the approach to the other.

## Conclusions

Early recognition and aggressive management remain the cornerstones of successful DKA treatment. However, clinicians must remain vigilant for rare but serious complications such as cerebral and pulmonary oedema, particularly in adult patients.

This case illustrates the complex, interconnected pathophysiology underlying critical illness in DKA. The development of both cerebral and non-cardiogenic pulmonary oedema in an adult is a rare but serious event. It highlights the shift in our understanding of cerebral oedema from a purely osmotic phenomenon to a multifactorial process involving cerebral hypoperfusion and reperfusion injury. It also underscores the diverse mechanisms of pulmonary oedema in this setting, with our patient's course most consistent with a permeability-mediated aetiology.

Crucially, the management of this patient required a delicate, individualised balance, navigating the sometimes conflicting priorities of fluid resuscitation, osmotic control, and organ support. This case reinforces the need for a high index of suspicion for these complications in adults with DKA and for a treatment strategy that integrates evolving pathophysiological concepts with meticulous clinical monitoring.
